# Impact of physical activity on monocyte subset CCR2 expression and macrophage polarization following moderate intensity exercise

**DOI:** 10.1016/j.bbih.2019.100033

**Published:** 2019-12-27

**Authors:** Anson M. Blanks, Thomas T. Wagamon, Lindsay Lafratta, Mabel G. Sisk, Morgan B. Senter, Lauren N. Pedersen, Natalie Bohmke, Attiya Shah, Virginia L. Mihalick, R. Lee Franco

**Affiliations:** Department of Kinesiology and Health Sciences, College of Humanities and Sciences, Virginia Commonwealth University, Richmond, VA, United States

**Keywords:** Monocytes, Macrophages, CCR2, Physical activity, Exercise

## Abstract

Coronary artery disease (CAD) is an immune-mediated disease in which CCR2 attracts classical, intermediate, and non-classical monocytes to the arterial intima where they differentiate to macrophages. Balance between pro-inflammatory M1 and anti-inflammatory M2 macrophages contributes to CAD prevention. Moderate to vigorous intensity physical activity (MVPA) elicits an immune response and reduces the incidence of CAD, however, the impact of prior MVPA on monocyte subset CCR2 expression and macrophage polarization following acute exercise is unknown.

**Purpose:**

To determine the impact of physical activity status on monocyte subset CCR2 surface expression and macrophage polarization in response to an acute bout of moderate intensity cycle ergometry.

**Methods:**

24 healthy women and men (12 high physically active [HIACT]: ≥1500 METmin/wk MVPA & 12 low physically active [LOACT]: <600 METmin/wk MVPA) underwent an acute moderate intensity (60% VO_2peak_) bout of cycle ergometry for 30 ​min. Blood samples were collected prior to (PRE), immediately (POST), 1 ​h (1H), and 2 ​h (2H) following exercise. Monocyte CCR2 and macrophage CD86 (M1) and CD206 (M2) were analyzed by flow cytometry.

**Results:**

Intermediate monocyte CCR2 decreased in response to exercise in the HIACT group (PRE: 11409.0 ​± ​1084.0 vs. POST: 9524.3 ​± ​1062.4; p ​= ​0.034). Macrophage CD206 was lower in the LOACT compared to the HIACT group at 1H (HIACT: 67.2 ​± ​5.6 vs. LOACT: 50.1 ​± ​5.2%; p ​= ​0.040). Macrophage CD206 at 1H was associated with both PRE (r ​= ​0.446, p ​= ​0.043) and POST (r ​= ​0.464, p ​= ​0.034) non-classical monocyte CCR2.

**Conclusion:**

These data suggest that regular moderate to vigorous physical activity positively impacts both monocytes and macrophages following acute moderate intensity exercise and that this impact may contribute to the prevention of coronary artery disease.

## Highlights

• Exercise reduces intermediate monocyte CCR2 in high physically active subjects.
• M2 macrophages are preserved in high active and reduced in low active subjects.
• Exercise increases the percentage of classical monocytes.
• Non-classical monocyte CCR2 expression is associated with macrophage polarization.

## 1 Introduction

Coronary artery disease (CAD), the most common form of cardiovascular disease, is the leading cause of death in developed nations (Mozaffarian et al., 2015). Although preventative pharmacological interventions have been shown to reduce the incidence of CAD, to date, the most effective prevention strategy is habitual physical activity (Agarwal, 2012).

CAD is a pro-inflammatory immune-mediated disease in which chemokines and chemokine receptors, such as C–C chemokine ligand 2 (CCL2, also known as MCP-1) and C–C chemokine receptor (CCR2), are critical for the attraction of various leukocytes to the arterial intima (Moore and Tabas, 2011). During an acute pro-inflammatory immune response, such as following antigen activation or tissue damage, one of the first and most highly recruited cell types are monocytes (Moore and Tabas, 2011). Monocytes are divided into three phenotypically and functionally distinct subsets based on surface expression of CD14 and CD16 receptors (Mukherjee et al., 2015). Under homeostatic conditions, classical monocytes (CD14^++^CD16^−^) are released daily from bone marrow (Patel et al., 2017) and are anti-inflammatory due to the high level production of the hallmark anti-inflammatory cytokine IL-10 (Wong et al., 2011). The intermediate (CD14^++^CD16^+^) and non-classical (CD14^Low^CD16^++^) subsets are considered to be more mature pro-inflammatory monocytes due to the production of cytokines IL-1β and TNF-α (Mukherjee et al., 2015; Ong et al., 2018). In addition, intermediate and non-classical monocytes are responsible for the ingestion of debris, as well as extra-cellular matrix breakdown necessary for tissue repair (Mukherjee et al., 2015; Wong et al., 2011; Ong et al., 2018). Once within the tissue, monocytes differentiate into macrophages, which are broadly classified into pro-inflammatory M1 or anti-inflammatory M2 phenotypes (Moore and Tabas, 2011; Italiani and Boraschi, 2014). M1 phenotypes are elicited in response to pro-inflammatory antigen activation and/or a pro-inflammatory cellular microenvironment and this macrophage phenotype is responsible for pathogen destruction via the oxidative burst, as well as extracellular matrix breakdown following damage (Italiani and Boraschi, 2014; Gonzalez-Dominguez et al., 2016). In the absence of pro-inflammatory activation or in presence of an anti-inflammatory microenvironment, macrophages develop an anti-inflammatory M2 phenotype, which is responsible for immunosurveillance and collagen deposition (Italiani and Boraschi, 2014; Boyette et al., 2017). Under normal regulation, proper M1/M2 macrophage balance contributes to CAD prevention (Moore et al., 2013). However, an increase in the M1 phenotype leads to greater production of pro-inflammatory cytokines, eliciting a microenvironment that switches the M2 phenotype toward the M1 phenotype, which results in the skewing of macrophage balance toward the M1 phenotype (Moore et al., 2013). This process precipitates sustained low-grade inflammation and accumulation of lipid rich macrophages, which eventually progress into foam cells and atherosclerotic lesions (Moore and Tabas, 2011).

Moderate to vigorous intensity physical activity (MVPA) is known to elicit a transient immune response, which includes monocytosis (Shinkai et al., 1992). This response increases monocyte turnover (Claycombe et al., 2008), increasing anti-inflammatory monocytes in circulation (Timmerman et al., 2008). These anti-inflammatory monocytes promote an anti-inflammatory microenvironment which contributes to the maintenance of M1/M2 macrophage balance in tissue, thereby reducing the incidence of CAD (Moore et al., 2013; Wattananit et al., 2016). Regardless of activity status, acute MVPA induces an immune response and repeated bouts of activity have been shown to alter leukocyte phenotype and function (Ruffino et al., 2016). CCR2 is responsible for monocyte chemotaxis to tissue (Bartoli et al., 2001) and recent investigations have shown a significant role of CCL2-CCR2 interaction in M2 macrophage polarization (Sierra-Filardi et al., 2014; Deci et al., 2018). To date, only one study has examined the monocyte CCR2 response to acute aerobic exercise (Okutsu et al., 2008). While investigators did not observe a significant change in monocyte CCR2 expression, it is important to note that the study did not examine potential physical activity related differences among participants, nor did the study differentiate CCR2 expression amongst monocyte subsets. Importantly, aerobic training studies in both human and animal models have shown the beneficial impact of exercise on macrophage polarization in skeletal muscle (Walton et al., 2019) and adipose tissue (Kawanishi et al., 2010). However, to our knowledge, there are currently no studies that have investigated the potential role of CCR2 on circulating primary monocytes in human macrophage polarization following a single bout of aerobic exercise. In addition, the impact of habitual MVPA on monocyte subset CCR2 expression and macrophage polarization following an acute bout of exercise is unknown. Although habitual MVPA has unquestionable protective benefits against the development of CAD, the mechanisms responsible remain elusive. Therefore, the purpose of this investigation was to determine the impact of prior moderate to vigorous physical activity on monocyte subset CCR2 surface expression and macrophage polarization following an acute bout of moderate intensity cycle ergometry.

## 2 Methods

### 2.1 Experimental design

Twenty four healthy women and men volunteered to participant in the study. Physical activity levels were determined by scores from the long form International Physical Activity Questionnaire (IPAQ) (Hagstromer et al., 2006). Inclusion criteria consisted of normal body mass index (18.5–24.9 ​kg/m2), 18–30 years of age, and normal percentage of body fat (males: 3–25%; females: 10–30%) (Jeukendrup and Gleeson, 2010). Exclusion criteria consisted of tobacco use and use of medications that may have impacted metabolism. In order to limit the impact of sex hormones on monocytes, submaximal testing was performed during the early follicular phase (first 7 days) of the menstrual cycle for all female participants. All participants were instructed to limit their physical activity 3 days prior to testing. Moderate to vigorous physical activity (MVPA) was used to classify subjects into high physically active (HIACT: n ​= ​12; MVPA: ≥1500 MET min/wk) and low physically active (LOACT: n ​= ​12; MVPA: <600 MET min/wk) groups. Groups were chosen to differentiate between sufficient levels of physical activity for optimal health benefits (HIACT) and some activity but not sufficient for health benefits (LOACT), while simultaneously excluding sedentary individuals (Sjöström et al., 2006). Macrophage phenotypes exist on a broad spectrum, with M1 and M2 phenotypes representing pro-inflammatory and anti-inflammatory extremes, respectively. Healthy young men and women tend to have homogenous immune profiles, regardless of fitness status (Beiter et al., 1985). Therefore, in order to clearly determine the potential impact of acute exercise on macrophage polarization we chose to focus on M1/M2 macrophage extremes. Study procedures were approved by the Virginia Commonwealth Institutional Review board (IRB#HM200008223) and all participants signed an informed consent, volunteering to participate in the study.

### 2.2 Body composition testing

Participants arrived at the Virginia Commonwealth University Exercise Physiology Research Laboratory (EPRL) between 7:00–8:00 a.m. following an overnight fast, where height and weight were assessed. Body composition was then assessed using air displacement plethysmography (BodPod, Cosmed, Rome, Italy) according to the manufacture’s recommendations. Briefly, the BodPod was calibrated daily according to manufacturer’s instructions and participants were instructed to wear minimal tight fitting clothing, remove all jewelry, and place their hair under a swim cap. Participants were then seated within the BodPod and two consistent (±1%) body volume measurements were taken. Measurements were entered into the manufacturer’s software and percentage of fat mass was calculated using the Siri equation (Siri, 1961).

### 2.3 Peak graded exercise testing

Following analysis of body composition, participants were fitted with a chest strap heart rate monitor (Polar Electro Inc., New York, USA) and asked to sit quietly for 5 ​min. Resting heart rate was recorded and blood pressure was manually assessed by an experienced technician using a sphygmomanometer and stethoscope. Standard gas and volume for the metabolic measurement system (TrueOne 2400, ParvoMedics, UT, USA) were calibrated daily according to manufacturer’s instructions. The acceptable percent change for calibrations was <1%. Participants were seated on an electronically braked cycle ergometer (Ergoselect 100, Ergoline, Bitz, Germany) and connected to the metabolic measurement system in order to perform gas exchange analysis. Before beginning exercise, the test protocol was explained and a resting blood lactate measurement was obtained from a finger stick blood sample using a blood lactate analyzer (Lactate Scout +, EKF Diagnostics, Cardiff, England). Three minutes of pre-exercise data was collected in order to ensure gas exchange measurements were within acceptable physiological ranges (Mezzani, 2017). Following the rest period, participants entered a low intensity warm-up stage where they were instructed to pedal at a cadence of 50–100 RPM against a constant workload (Men: 50W; Women: 25W) (Denadai et al., 2005). After completion of the warm-up stage, the workload was consistently increased (Men: 25 ​W/min; Women: 15 ​W/min) until volitional fatigue (Zhang et al., 1991; Albouaini et al., 2007). Peak effort during the test was determined if a participant reached three of the following criteria: peak heart rate ±10 beats of age predicted maximal heart rate (220-age), a rating of perceived exertion ​≥ ​17 on the Borg scale, blood lactate ​≥ ​8 ​mmol/L, and a respiratory exchange ratio ​> ​1.1 (Edvardsen et al., 2014).

### 2.4 Submaximal exercise testing

Participants were asked to return to the EPRL at least 3 days following peak graded exercise testing. Again, participants were instructed to limit their physical activity 3 days prior to testing and to fast overnight. Participants’ weight was assessed and they were fitted with a chest strap heart rate monitor. In order minimize the impact of stress hormones and cardiovascular parameters on immune function, prior to exercise participants were seated and asked to rest quietly for 30 ​min (Hill et al., 2008; Gu et al., 1999; Riou et al., 2007). Resting heart rate was then recorded and blood pressure was manually assessed. A pre-exercise blood sample (PRE) was obtained from an antecubital vein following standard venipuncture guidelines (In:WHO guidelines, 2010). Venous blood was obtained in two 10 ​mL blood collection tubes coated with sodium heparin and one 10 ​mL serum separator tube (SST) (BD Vaccutainer, Becton, Dickinson and Company, NJ, USA). The exercise testing procedure was explained to the participants and they were then seated on the same cycle ergometer and connected to the same metabolic measurement system used for peak exercise testing. Three minutes of resting gas exchange data was collected. Participants then performed a 3 ​min warm-up period identical to the peak exercise test warm-up. The warm-up workload (Men: 25 ​W/min; Women: 15 ​W/min) was subtracted from the measured workload at 60% of VO_2peak_ and the difference was divided by 5. This calculation provided a value that was used to increase the workload in equal increments each minute following warm-up until participants reached a workload corresponding to 60% of VO_2peak_. Participants maintained this workload for 25 ​min. If necessary, participants’ workload was adjusted in order to maintain 60% of VO_2peak_. Blood lactate was measured from a finger stick blood sample every 5 ​min to ensure participants were below lactate threshold.

### 2.5 Sample processing and whole blood flow cytometry staining

Immediately following completion of the submaximal exercise test, venous blood was obtained in two 10 ​mL sodium heparin tubes and one 10 ​mL SST tube (POST). Subsequent to POST venipuncture, participants were asked remain fasted while sitting in the EPRL, and to avoid engaging in activities that may have been mentally stressful (exam studying, work deadline, etc.) in order to limit the impact of stress on immune function. To assess the time course of the monocyte response, additional venous blood samples identical to PRE and POST were obtained 1 ​h (1H) and 2 ​h (2H) following POST measures. PRE and POST blood samples were processed together. Briefly, 200 ​μL of whole blood was removed from each tube and placed into 2 ​mL microcentrifuge tubes (Safe-lock, Eppendorf, Hamburg, Germany). Whole blood was washed once using 1.8 ​mL of freshly prepared flow cytometry staining buffer (1× PBS ​+ ​4% FBS). Blood samples were centrifuged at 1000 × G for 10 ​min and supernatant was aspirated and discarded. Careful attention was paid not to disturb the buffy coat. Next, 1.8 ​mL of freshly prepared commercial lyse/fix buffer (BD Phosflow Lyse/fix, Becton, Dickinson and Company) was added and blood was incubated in a water bath at 37 ​°C for 10 ​min in order to lyse erythrocytes. Tubes were centrifuged at 600 × G for 10 ​min, supernatant was decanted and discarded, and cells were again washed with 1 ​mL of staining buffer. Supernatant was decanted and cells were suspended in 1 ​mL of freshly prepared commercial permeabilization buffer (BD perm/wash buffer, Becton, Dickinson and Company) and incubated at room temperature for 20 ​min. Cells were centrifuged for 10 ​min at 600 × G and supernatant was decanted and discarded. Cells were washed once using 1 ​mL of permeabilization buffer and suspended in 200 ​μL of permeabilization buffer. In order to block non-specific binding of Fcγ receptors on myeloid cells, 5 ​μL of commercial Fc block (Human TruStain FxX, Biolegend, CA, USA) was added to each tube and tubes were incubated at room temperature for 10 ​min. To identity monocytes, antibodies against CD14 (FITC conjugated anti-human antibody, clone: M5E2, 0.5 μL/test, Biolegend), CD16 (APC conjugated anti-human antibody, clone: 3G8, 5 μL/test, Biolegend), CCR2 (PE conjugated anti-human antibody, clone: K036C2, 2.5 μL/test, Biolegend) were added at optimal concentrations as determined by previous titration experiments and incubated at room temperature protected from light for 1 ​h. Cells were washed twice using 3 ​mL of permeabilization buffer, suspended in 500 ​μL of permeabilization buffer, and stored at 4 ​°C protected from light until flow cytometry analysis. Flow cytometry staining was repeated in an identical fashion for 1H and 2H blood samples. All analyses were performed ≤3 days subsequent to processing for all whole blood samples.

### 2.6 Macrophage culture & flow cytometry staining

Following the removal of blood used for flow cytometry staining, 18 ​mL of heparinized blood was carefully layered onto 16 ​mL of room temperature Hisotopaque 1077 (Sigma-Alrdich, MO, USA). Samples were centrifuged at 600 × G for 20 ​min. The top plasma layer was carefully aspirated and stored at −80 ​°C. The PBMC layer was collected using a micropipette and washed twice using sterile PBS at 1000 × G for 10 ​min. In order to facilitate platelet removal, cells were washed using PBS +1% FBS and centrifuged at 200 × G for 15 ​min. Cells were washed once more with sterile PBS at 1000 × G for 10 ​min. PBMCs were counted and 200 ​μL of cell suspension was plated in duplicate wells at a concentration of 5 ​× ​10^6^ ​cells/mL in a 48 well tissue culture treated microplate (Corning Incorporated, MA, USA). Cultures were placed into an incubator at 37 ​°C with 5% CO_2_ for 2 ​h in order to allow monocytes to adhere. Cultures were removed from the incubator, the cell culture supernatant was aspirated and discarded, and plates were washed with sterile PBS at 1000 × G for 5 ​min 200 ​μL of 37 ​°C complete culture media (DMEM+1% pen-strep+20% autologous serum) was added to each well and plates were returned to the incubator. Culture media was aspirated and replaced with complete media every 2–3 days for a total of 7 days.

Following the 7 day culture, cell cultures were washed with PBS and macrophages were released from the plastic by incubating wells with 300 ​μL of cell detachment solution (Accutase, Innovative Cell Technologies, Inc., CA, USA) at room temperature for 25 ​min (Davies et al., 2017). Supernatant was collected and transferred to 2 ​mL microcentrifuge tubes. In order to assess cell viability, macrophages were stained using a viability dye (Zombie Aqua Fixable Viability Kit, Biolegend) at a 1:500 concentration for 20 ​min. Cells were washed once using staining buffer and stained using antibodies against CCR2, the M2 marker CD206 (PE/Cy5 conjugated anti-human CD206 (MMR) antibody, clone: 15–2, 5 μL/test, Biolegend), and the M1 marker CD86 (Alexa Fluor 647 conjugated anti-human CD86 antibody, clone: IT2.2, 2.5 μL/test, Biolegend) in identical fashion as whole blood samples. Macrophages were analyzed immediately following antibody staining.

### 2.7 Flow cytometry analysis

**Fig. 1:**
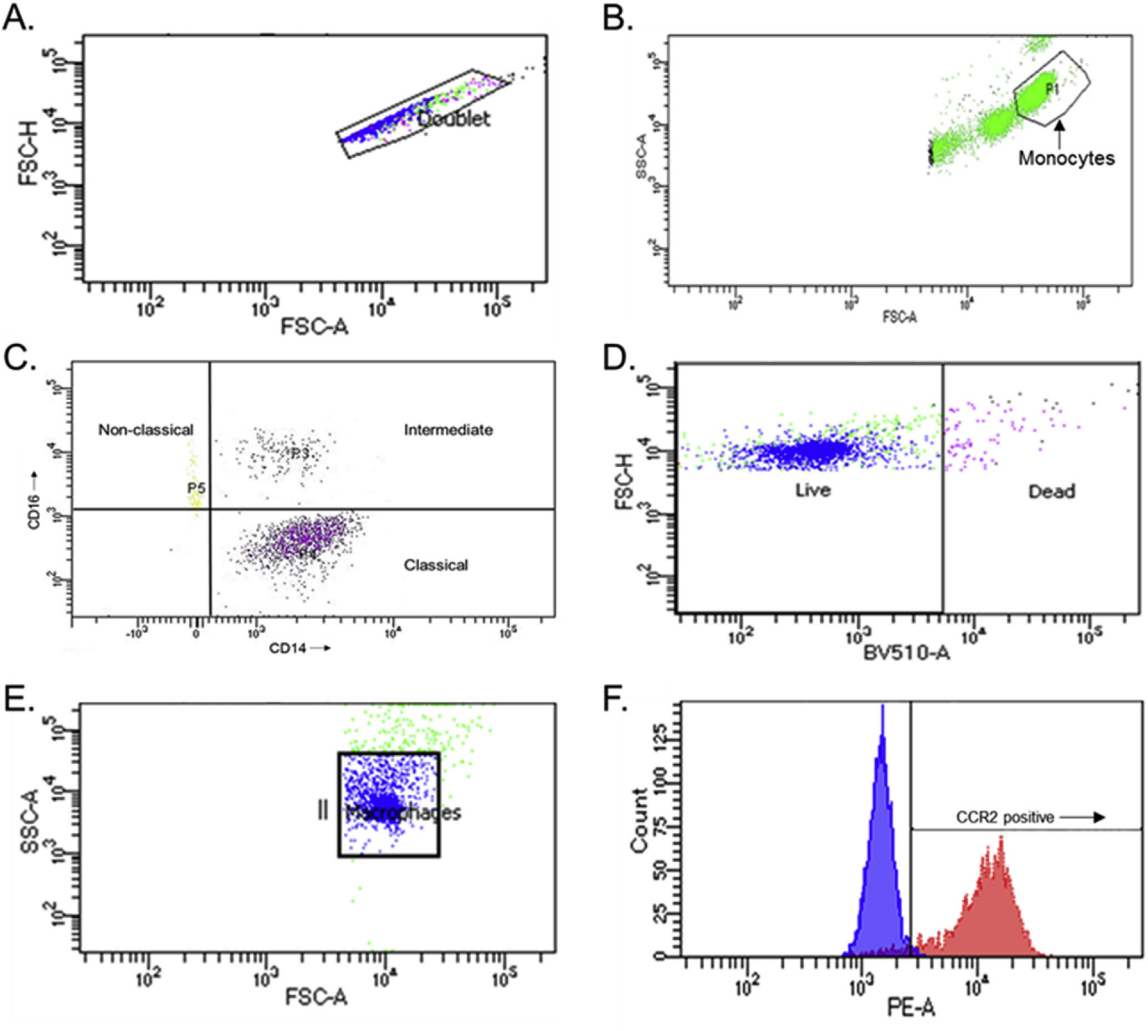
Doublet cells were gated out using forward scatter height (FSC–H) and forward scatter area (FSC-A) (A). Monocytes were determined by side scatter area (SSC-A) and FSC-A (B). Monocyte subsets were gated based on expression of CD14 and CD16 (C). Macrophage viability (D) was assessed and macrophages were gated by SSC-A and FSC-A (E). Monocyte CCR2 and macrophage CCR2, CD86, and CD206 were assessed using histogram analysis (F). Fluorescence minus one control samples (purple) were used to set gates for positive receptor expression (red). (For interpretation of the references to colour in this figure legend, the reader is referred to the Web version of this article.)

All flow cytometry analyses were performed on a FACSCelesta (Becton, Dickinson and Company, NJ, USA) within the John Ryan Laboratory at VCU. Flow cytometer setup and tracking was performed daily. Flurochrome compensation was performed using unstained controls and compensation beads (Ultracomp ebeads compensation beads, ThermoFisher, MA, USA) stained with the antibodies being used in the experiment. Doublet cells were gated out using a dot plot display of forward scatter area versus forward scatter height (Fig. 1A). Following doublet gating, monocytes were initially determined and gated based on forward and side light scatter profiles and 2000 events were collected. An intracellular staining buffer (Intracellular Staining Permeabilization Wash Buffer, Biolegend, San Diego, CA) for markers not included in this investigation was used. When compared to standard flow cytometry staining buffer (1× PBS +4% FBS), this buffer altered the cell light scatter profile but did not impact receptor expression (pilot data not shown). In order to ensure the inclusion of all monocyte events, the monocyte scatter gate was widened (Fig. 1B). Monocytes were confirmed and gated into subset quadrants using a dot plot of CD14 versus CD16 (Fig. 1C). Macrophages were gated in a similar fashion. Briefly, doublet cells were gated out, a gate was set for live cells (Fig. 1D) and macrophages were gated based on scatter profile (Figs. 1E), and 2000 events were collected. Adequate blocking of Fcγ receptors was assessed using appropriately matched isotype controls (Biolegend) and receptor positivity was determined using fluorescence minus one controls. Histogram analysis was then performed to analyze receptor expression within each monocyte and macrophage subset (Fig. 1F). Monocyte and macrophage expression is reported as mean fluorescent intensity in arbitrary units of fluorescence (AUF).

### 2.8 Statistical analysis

Demographics of the study participants were compared using descriptive statistics and independent samples t-tests. Due to the influence of blood pressure on monocyte adhesion and diapedesis, mean arterial pressure (MAP) was analyzed as a covariate (Riou et al., 2007; Tropea et al., 1996). Two-way analysis of covariance factorial (group x time ANCOVAs) with Bonferroni adjustments were used to determine differences in monocyte subset CCR2 expression, percentage of monocytes positively expressing CCR2 (CCR2^+^), and percentage of monocyte subsets between and within groups (HIACT & LOACT) across all time points. Two-way (group x time) ANCOVAs were used to determine differences in macrophage CD206, CD86, CCR2 expression, and percentage of macrophages positively expressing the aforementioned markers (CD206^+^, CD86^+^, CCR2^+^). Effect sizes (partial eta squared [η_Pˆ2]) are reported for the interaction terms of the ANOVA, where values of 0.01, 0.06, and 0.14 correspond to small, medium, and large effects, respectively (Cohen, 1988). Statistical analyses were performed with SPSS Version 24 software (IBM) and data are presented as mean ​± ​standard error of the mean (SEM). The level of significance for all tests was set a priori at α ​≤ ​0.05.

## 3 Results

Participant demographics are presented in Table 1. By design, a statistically significant difference was observed in physical activity. As physical activity has been strongly associated with VO_2peak_ (Schembre and Riebe, 2011), a significantly different VO_2peak_ was observed between the HIACT and LOACT groups. No other significant differences were observed between groups.

**Table 1:** Participant demographics for high physically active (HIACT) and low physically inactive (LOACT) groups. MVPA (Moderate-to-vigorous physical activity), MAP (Mean arterial pressure). Data are presented as the mean ​± ​standard error of the mean. *p ​< ​0.05 between groups; Independent samples *t*-test.

Variable	HIACT (n ​= ​12)	LOACT (n ​= ​12)	p-value
Sex (F/M)	6/6	6/6	n/a
Age (yrs)	23.8 ​± ​0.7	22.8 ​± ​0.9	0.396
Height (cm)	170.6 ​± ​2.3	170.1 ​± ​3.6	0.900
Weight (kg)	65.5 ​± ​2.5	64.2 ​± ​3.6	0.769
Body Mass Index (kg/m^2^)	22.5 ​± ​0.4	22.4 ​± ​0.6	0.932
Body Fat (%)	16.3 ​± ​1.9	18.6 ​± ​1.9	0.395
MVPA (METmin/wk)	3848.3 ​± ​593.3	378.1 ​± ​65.5	<0.001*
VO_2peak_ (L min^−1^)	3.0 ​± ​0.3	2.1 ​± ​0.2	0.007*
VO_2peak_ (mL kg min^−1^)	45.0 ​± ​2.5	32.5 ​± ​1.6	<0.001*
MAP (mmHg)	84.9 ​± ​2.4	89.9 ​± ​1.9	0.117

**Blanks, AM** Physical Activity, Monocyte CCR2, and M1/M2 Macrophages.

### 3.1 Monocyte CCR2 expression & subset response

**Fig. 2:**
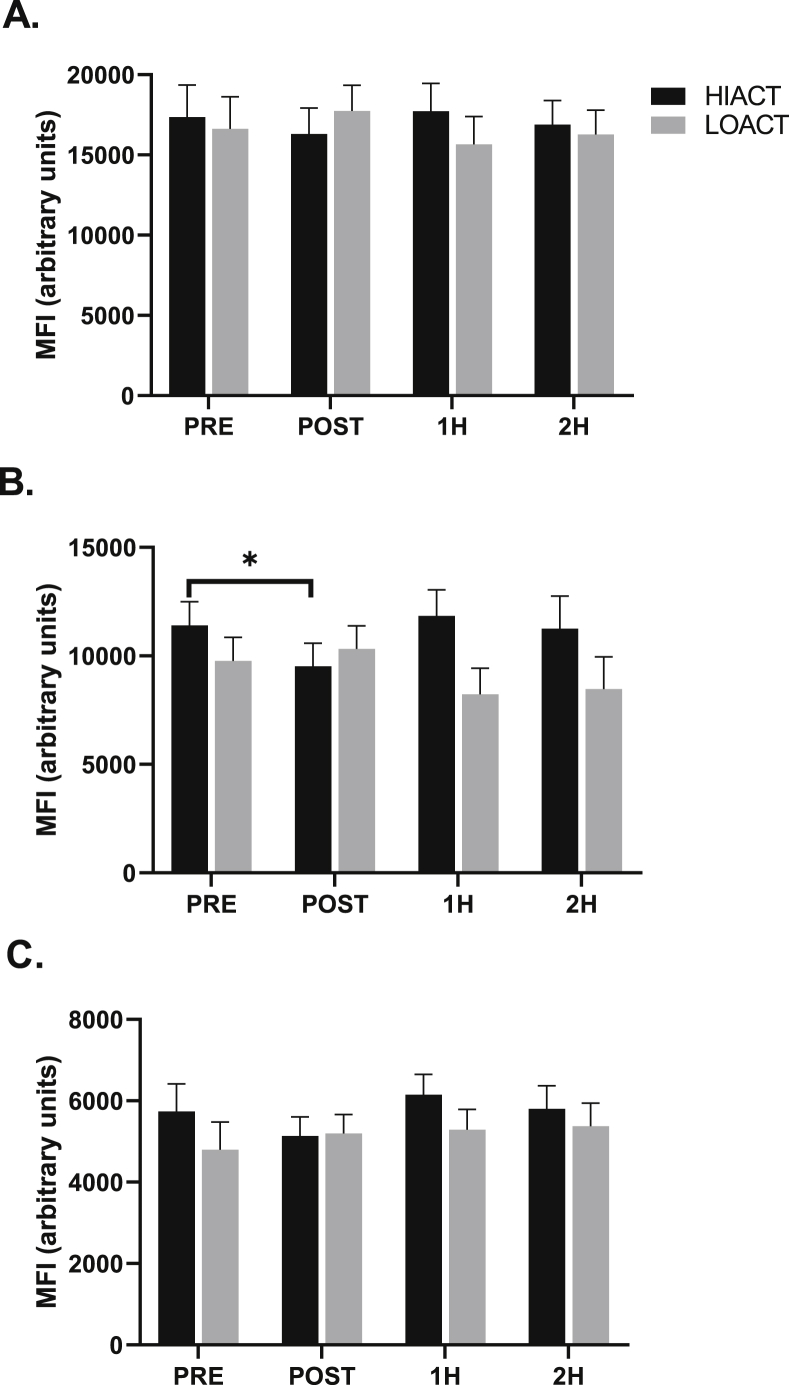
Time course of the mean fluorescent intensity (MFI) of CCR2 on the classical (A), intermediate (B), and non-classical (C) monocyte subsets in high physically active (HIACT) and physically low active (LOACT) individuals. 2 ​× ​4 repeated measures ANCOVA. *p ​< ​0.05 PRE vs. POST within HIACT group; 2 ​× ​4 repeated measures ANCOVA.

**Fig. 3:**
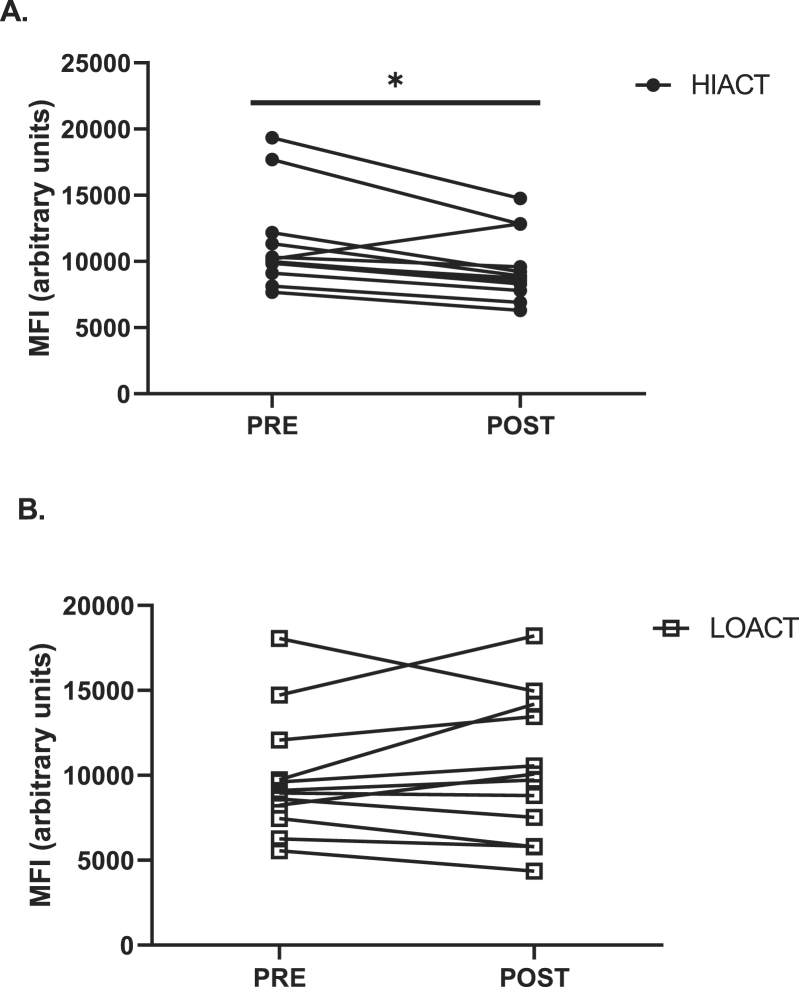
Individual values of intermediate monocyte CCR2 MFI in HIACT (B) and LOACT (C) groups. *p ​< ​0.05 PRE vs. POST within HIACT group; 2 ​× ​4 repeated measures ANCOVA.

**Fig. 4:**
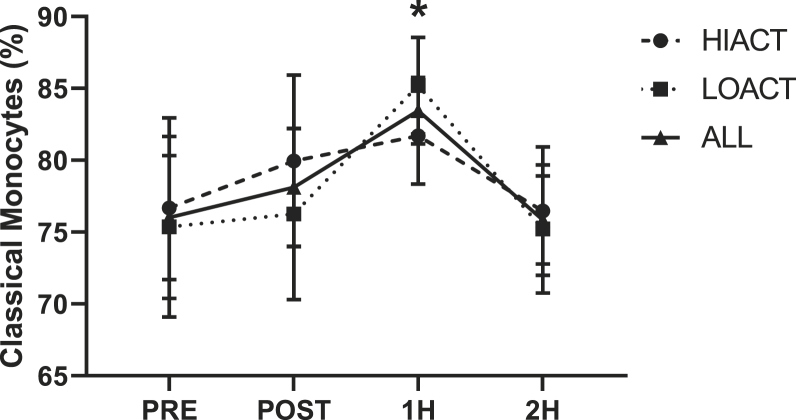
Time course of the percentages of classical monocytes in high physically active (HIACT), low physically active (LOACT), and both groups analyzed together (ALL). *p ​< ​0.05 1H vs. 2H time effect for ALL; 2 ​× ​4 repeated measures ANCOVA.

Pre-exercise CCR2 expression was not different between groups in any monocyte subset. Classical (Fig. 2A) and non-classical (Fig. 2C) CCR2 expression was not changed at any time point in either group following exercise (p ​> ​0.05). A group by time effect was observed for intermediate monocyte CCR2 expression (p ​= ​0.040, η ​= ​0.123) in response to exercise (Fig. 2B). Individual values for PRE and POST intermediate monocyte CCR2 expression are presented in Fig. 3A & B. In the HIACT group intermediate CCR2 expression was reduced immediately post-exercise (PRE: 11409.0 ​± ​1084.0 vs. POST: 9524.3 ​± ​1062.4 AUF; p ​= ​0.034) (Fig. 3A). Intermediate CCR2 expression returned to baseline at 1H (1H: 11847.0 ​± ​1191.6 AUF) (Fig. 2B). Intermediate CCR2 expression was not impacted by exercise in the LOACT group (p ​> ​0.05) (Fig. 2B). The percentage of CCR2^+^ monocytes was not changed in any monocyte subset in either group following exercise (p ​> ​0.05). For all subjects as whole, a time effect (p ​= ​0.020, η ​= ​0.14) was observed for the percentage of classical monocytes (1H: 83.5 ​± ​2.3 vs. 2H: 75.8 ​± ​3.1%, p ​= ​0.008) (Fig. 4).

### 3.2 Macrophage polarization

**Fig. 5:**
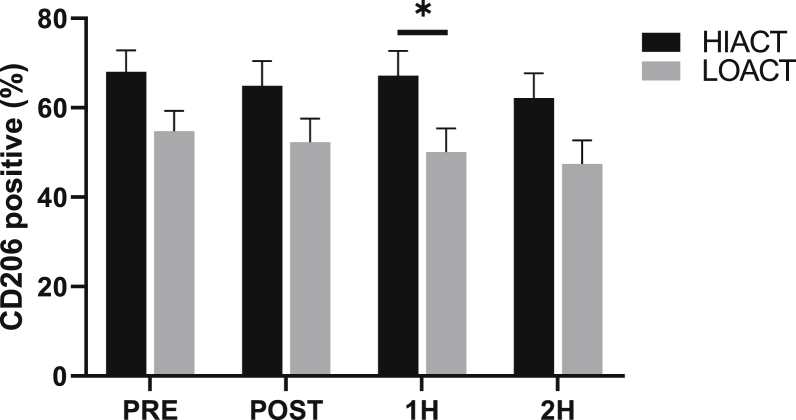
Time course of the percentage of macrophages expressing CD206 in high physically active (HIACT) and low physically active (LOACT) individuals. *p ​< ​0.05 between groups; 2 ​× ​4 repeated measures ANCOVA.

No pre-exercise differences were observed between groups in the percentage of CD86^+^, CD206^+^, or CCR2^+^ macrophages (p ​> ​0.05). Additionally, no difference was observed in receptor expression at PRE. A group by time effect was not observed in macrophage polarization (p ​> ​0.05); however, a significant difference was found between groups (p ​= ​0.049, η ​= ​0.199) in the percentage of CD206^+^ macrophages at 1H (HIACT: 67.2 ​± ​5.6 vs. LOACT: 50.1 ​± ​5.2%; p ​= ​0.040) (Fig. 5). No differences were observed in the M1/M2 ratio at any time point (p ​> ​0.05).

### 3.3 Relationships between monocytes & macrophages

When all participants were analyzed together, macrophage CD206 expression at 1H was positively associated with non-classical monocyte CCR2 expression at PRE (r ​= ​0.446, p ​= ​0.043) as well as POST (r ​= ​0.464, p ​= ​0.034). The percentage of CCR2^+^ non-classical monocytes at PRE was negatively associated with macrophage CD86 expression at PRE (r ​= ​-0.415, p ​= ​0.028). Immediately post-exercise, macrophage CD86 expression was negatively associated with the percentages of classical (r ​= ​-0.436, p ​= ​0.33) and non-classical (r ​= ​-0.455, p ​= ​0.025) CCR2^+^ monocytes at POST. When expressed as a percentage of total monocytes, the percentage of the classical subset at POST was negatively associated with M1/M2 macrophage ratio at POST (r ​= ​-0.405, p ​= ​0.049), the percentage of the intermediate subset at POST was negatively associated with M1/M2 macrophage ratio at 2H (r ​= ​0.437, p ​= ​0.042), and the percentage of the non-classical subset at PRE was associated with M1/M2 macrophage ratio at PRE (r ​= ​0.408, p ​= ​0.048).

## 4 Discussion

The purpose of the present study was to determine if monocyte subset CCR2 surface expression and macrophage polarization in response to an acute bout of moderate intensity exercise are different between high physically active compared to low physically active individuals. Findings of the present study demonstrate that an acute bout of exercise elicits a monocyte response in both high and low physically active individuals. Although acute exercise elicits responses in both high and low active individuals, intermediate monocyte CCR2 is reduced and macrophage CD206 is unchanged in highly active individuals, as compared to low active individuals. To our knowledge, this is first study to demonstrate that monocyte CCR2 expression and macrophage polarization responses to a single session of moderate intensity exercise are beneficially impacted by high levels of prior physical activity.

The surface expression of CCR2 on the intermediate monocyte subset was reduced immediately post-exercise in the HIACT group. Previous investigations have consistently shown that exercise at or above 60% of VO_2peak_ in young healthy individuals causes an increase in plasma cortisol concentrations above resting levels (Budde et al., 2015). Although cortisol levels were not measured in the current investigation, a previous investigation of the monocyte CCR2 response to exercise showed that incubation of monocytes with post-exercise serum led to a cortisol dependent increase in CCR2 surface expression (Okutsu et al., 2008). Therefore, in the LOACT group it is plausible that exercise-induced cortisol did in fact increase CCR2 expression, however this elevation may have been balanced by the ligand-receptor internalization that occurs when CCL2 binds to CCR2 (Volpe et al., 2012). Acute increases in cortisol are necessary for a proper immune response (Dhabhar, 2002), however, chronic elevations of cortisol elicit immunosuppression via leukocyte desensitization to cortisol (Coutinho and Chapman, 2011). Although training status does not impact cortisol release in response to acute exercise in young healthy adults (Duclos et al., 1997), repeated exercise bouts may specifically reduce the response of pro-inflammatory monocytes to cortisol (Ehrchen et al., 2007), without immunosuppression due to the production of IL-6 and IL-10 that occur with exercise (Tsianakas et al., 2012; Pedersen et al., 2001; Cabral-Santos et al., 2019). Therefore, in the HIACT group, it does not appear that cortisol increased CCR2 expression and the observed post-exercise reductions in intermediate monocyte CCR2 expression were likely due to CCR2-CCL2 binding and internalization. Activated intermediate monocytes are pro-inflammatory in nature and contribute to the pro-inflammatory microenvironment which elicits monocytes to differentiate into pro-inflammatory M1 macrophages (Wong et al., 2011; Italiani and Boraschi, 2014). This relationship was evidenced by the positive association between the percentage of intermediate monocytes immediately post-exercise and M1/M2 macrophage ratio 2 ​h following exercise. Monocyte CCR2 binding to CCL2 stimulates chemotaxis along a chemical ligand gradient and receptor-ligand internalization acts to clear CCL2 from circulation, thereby reducing activation of additional cells in circulation (Volpe et al., 2012). As CCR2 expression has been shown to directly impact monocyte chemotaxis (Fantuzzi et al., 1999), the lower post-exercise intermediate monocyte CCR2 expression observed in the HIACT group blunts the acute pro-inflammatory response to exercise and likely contributes to reduced M1 macrophage polarization in tissue. Taken together, these data demonstrate a potential mechanism through which regular physical activity acts to prevent CAD.

The percentage of macrophages expressing the anti-inflammatory M2 marker, CD206, was greater in the HIACT group compared to the LOACT group 1 ​h following exercise. Although a group by time effect was not observed, the percentage of CD206 positive macrophages appeared to be lower following exercise in the LOACT group while remaining unchanged in the HIACT group. Sustained inflammation, creates a pro-inflammatory microenvironment which leads to pro-inflammatory activation of monocytes, preferential differentiation to pro-inflammatory M1 macrophages, and macrophage phenotype switching from M2 to M1 (Moore and Tabas, 2011; Moore et al., 2013). Together, these pro-inflammatory alterations skew macrophage balance towards the M1 phenotype, thereby leading to the pathogenesis and progression of CAD (Moore et al., 2013). Acute bouts of exercise have been shown to elicit an acute pro-inflammatory response which is necessary for muscle repair following exercise (Suzuki, 2018; Yang and Hu, 2018). This pro-inflammatory response is followed by an anti-inflammatory response which acts to quench inflammation (Suzuki, 2018). Although an exercise induced cytokine response has been observed in both trained and untrained individuals, the magnitude and time course is different (Schild et al., 2016). The cytokine microenvironment in which macrophages are exposed to will impact macrophage polarization (Wang et al., 2014) and although the cytokine response to exercise was not assessed in the current investigation, it is likely that a greater magnitude and more rapid time course of anti-inflammatory cytokine production led to a favorable anti-inflammatory microenvironment and preservation of M2 macrophage polarization in the HIACT group. whereas pro-inflammatory cytokines reduced M2 macrophage polarization in the LOACT group. In healthy individuals, exercise eventually leads to an anti-inflammatory response (Brown et al., 2015), which likely occurred at the 2 ​h time point in the LOACT group, thereby returning M2 macrophage polarization to baseline. Taken together, these data suggest that physical activity status positively impacts CAD risk by preserving anti-inflammatory M2 macrophage polarization following an acute bout of exercise.

In addition to its role in monocyte chemotaxis, CCR2 activation has been shown to play a role in macrophage polarization (Sierra-Filardi et al., 2014; Deci et al., 2018). Although CCR2 expression was not altered in response to exercise in classical and non-classical monocyte subsets, the percentage of CCR2^+^ classical monocytes was negatively associated with macrophage expression of the M1 marker, CD86. Under homeostatic conditions, classical monocytes are considered to be anti-inflammatory and contribute to an anti-inflammatory microenvironment, which elicits monocyte differentiation to the M2 macrophage phenotype (Mukherjee et al., 2015; Boyette et al., 2017; Jakubzick et al., 2013). The CCL2 response to muscle damage induced by acute exercise is equivocal (Lu et al., 2011; Peake et al., 1985). Although CCL2 was not assessed in the current investigation, none of the participants were trained cyclist, therefore participants in both groups likely experienced exercise-induced muscle damage and a subsequent increase in plasma CCL2 concentrations (Burt et al., 2012; Fredsted et al., 2008). Therefore, greater percentages of classical monocytes expressing CCR2 led to more monocytes being activated by CCL2, reduced M1 macrophage polarization, and a more favorable M1/M2 macrophage ratio as a result. Moreover, non-classical monocyte CCR2 expression at PRE and POST was positively associated with macrophage expression of the M2 marker, CD206. The percentage of pre-exercise CCR2^+^ non-classical monocytes was negatively associated with macrophage CD86 expression prior to exercise, further supporting the involvement of CCR2 in macrophage polarization. The pro-inflammatory nature of the non-classical monocyte subset is thought to be due to senescence (Ong et al., 2018). Aging is associated with increased CVD risk due to a heightened inflammatory status, known as “inflammaging”, which includes increased production of CCL2 (Franceschi et al., 2018; Antonelli et al., 2006). Increased CCL2 may be a compensatory mechanism to cope with the expansion of the non-classical monocyte subset, which express low levels of CCR2 (Ong et al., 2018). Recent investigations support the hypothesis that regular physical activity reduces inflammaging (Flynn et al., 2019). Although the mechanisms responsible for physical activity blunting of inflammaging are unclear, it is plausible to suggest that chronic physical activity slows monocyte aging by preserving CCR2 expression, thereby altering the phenotype of non-classical monocytes towards a less inflammatory phenotype similar to that of younger classical and intermediate monocytes. Taken together, these data demonstrate a significant role of CCR2 expression in macrophage polarization for all monocyte subsets. Moreover, these data suggest that physical activity may beneficially impact the relationship between monocyte CCR2 expression and macrophage polarization.

## 5 Conclusion

This is the first study to demonstrate the impact of physical activity on monocyte CCR2 expression and in-vitro macrophage polarization following an acute bout of moderate intensity exercise. Perhaps most importantly, the monocyte and macrophage responses to acute exercise appear to be different between high physically active and low active individuals. Although the current investigation examined monocyte/macrophage responses to a single bout of exercise, based on the study findings, it is likely that repeated bouts of moderate to vigorous physical activity would lead to long term adaptations in low physically active individuals and improve their monocyte/macrophage response, similar to that of high physically active individuals. Future studies are warranted to investigate the potential impact of repeated bouts of physical activity on the monocyte and macrophage response. Nonetheless, the findings from the current study suggest that physical activity positively impacts both monocytes and macrophages following acute moderate intensity exercise and more importantly, this impact may contribute to the prevention of coronary artery disease.

## Funding

This research did not receive any specific grant from funding agencies in the public, commercial, or not-for-profit sectors.
